# Distribution of *HFE *Gene Variants in Patients Undergoing Genetic Testing in Türkiye: A Retrospective Analysis of 643 Cases

**DOI:** 10.5152/eurasianjmed.2026.261390

**Published:** 2026-05-06

**Authors:** Ahmet Kablan, Abdullah Sezer, Abdüllatif Bakır, Ahmet Kürşad Güneş, Elifcan Taşdelen, Firdevs Dinçsoy Bir, Haktan Bağış Erdem, Hanife Saat, Harun Erdal, Mustafa Tarık Alay, Yusuf Coşkun

**Affiliations:** 1 Department of Medical Genetics, Ankara Etlik City Hospital, Ankara, Türkiye; 2 Division of Hematology, Department of Internal Medicine, Ankara Etlik City Hospital, Ankara, Türkiye; 3 Division of Gastroenterology, Department of Internal Medicine, Ankara Etlik City Hospital, Ankara, Türkiye

**Keywords:** Hereditary hemochromatosis, population genetics, Turkish population, Türkiye

## Abstract

**Background:**

Hereditary hemochromatosis (HH) is a common genetic disorder of iron metabolism, most frequently associated with pathogenic variants in the *HFE* gene. The distribution of *HFE* variants shows marked population-specific differences. This study aimed to evaluate the frequency and genotype distribution of major *HFE* variants in a large Turkish cohort.

**Methods:**

A total of 643 patients who underwent *HFE* gene sequencing at a single tertiary center were retrospectively analyzed. Genotype and allele frequencies of common *HFE* variants, including p.Cys282Tyr, p.His63Asp, and p.Ser65Cys, as well as rare variants were calculated. Clinical and biochemical parameters were not included, and the analysis focused exclusively on genetic frequency data.

**Results:**

Among the 643 patients, 424 (65.9%) were wild-type for *HFE*. The most frequent variant was H63D heterozygosity, detected in 166 patients (25.8%). p.Cys282Tyr homozygosity was identified in only 4 patients (0.6%). Compound heterozygosity for p.Cys282Tyr and p.His63Asp was observed in 1 patient, while another patient showed homozygous His63Asp with heterozygous p.Arg224Trp. In terms of allele frequencies, p.His63Asp was the most common with 0.183, followed by p.Cys282Tyr with a frequency of 0.012.

**Conclusion:**

The findings demonstrate a distinct *HFE* genetic profile in the Turkish population, characterized by a low frequency of p.Cys282Tyr and a high prevalence of p.His63Asp heterozygosity. These results highlight the importance of population-specific genetic data for accurate interpretation of *HFE* testing and HH risk assessment.

**Main Points**

- In this large Turkish cohort, p.His63Asp (H63D) was the most frequent *HFE* variant, predominantly in the heterozygous state, whereas the classical p.Cys282Tyr variant was rare.
- Homozygosity for p.Cys282Tyr was uncommon, confirming that the genetic profile of hereditary hemochromatosis (HH) in Türkiye differs substantially from Northern European populations.
- Population-specific allele frequency differences highlight the necessity of using local genetic data for accurate interpretation of *HFE* testing and HH risk assessment.

## Introduction

Hereditary hemochromatosis (HH) is an autosomal recessive disorder characterized by increased intestinal iron absorption, leading to progressive iron overload and potential damage to multiple organs, including the liver, pancreas, heart, and joints. If left untreated, HH may result in cirrhosis, diabetes mellitus, cardiomyopathy, and increased mortality. Clinical hemochromatosis is more common in males than females, and early diagnosis through genetic testing allows timely intervention and prevention of irreversible organ damage.^1^

**Figure 1:**
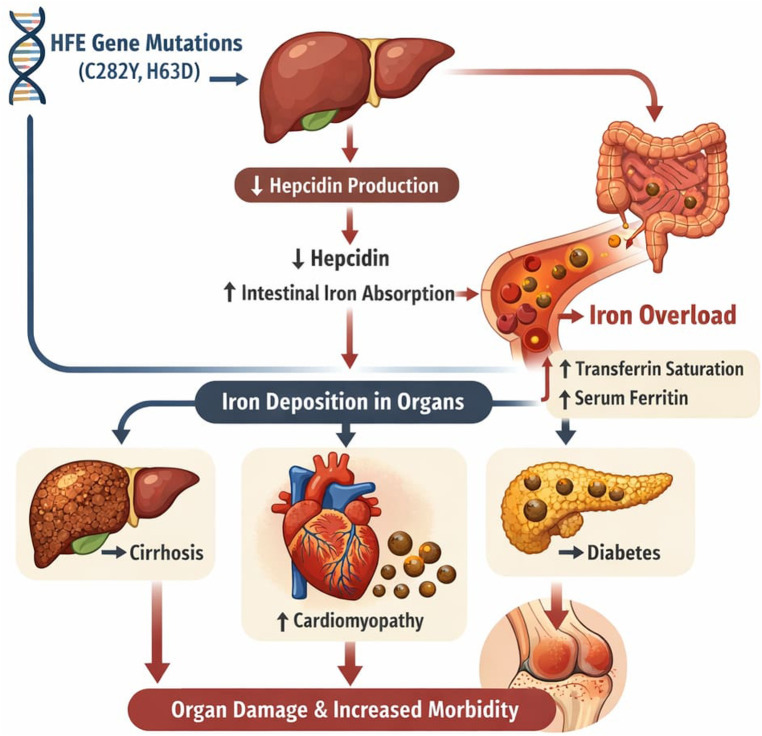
Schematic overview of iron homeostasis and the pathophysiology of hereditary hemochromatosis. Under physiological conditions, *HFE* positively regulates hepatic hepcidin expression. Pathogenic variants in *HFE* impair this regulatory mechanism, resulting in inappropriately low hepcidin levels, increased intestinal iron absorption, enhanced iron release from macrophages, elevated circulating iron parameters, and progressive iron accumulation in target organs.

The *HFE* gene is composed of 7 exons distributed over approximately 12 kb of genomic DNA. It spans 9609 bp on chromosome 6p21.3 and is located within the extended HLA class I region, flanked by histone gene clusters on both sides. Exon 1 encodes the signal peptide, while exons 2 through 4 correspond to the α1, α2, and α3 domains of the protein, respectively. The transmembrane region is encoded by exon 5, and the cytoplasmic tail is derived from the 5′ portion of exon 6, which also contains the native stop codon. Consequently, the mature *HFE* transcript consists of 6 exons. *HFE* is a positive upstream regulator of hepcidin, and pathogenic variants in *HFE* impair its interaction, leading to reduced hepatic hepcidin expression, increased intestinal iron absorption, enhanced iron release from macrophages, and progressive systemic iron overload (Figure 1).^2^

Among HH-related genetic variants, the c.845G>A (p.Cys282Tyr, C282Y) variant is considered the primary disease-causing mutation, particularly in individuals of Northern European ancestry. Homozygosity for p.Cys282Tyr accounts for the vast majority of clinically overt HH cases in these populations. Another common variant, c.187C>G (p.His63Asp, H63D) is widely distributed across different populations but exhibits lower penetrance and is usually associated with milder phenotypes or acts as a modifier in compound heterozygous states.^3^ The prevalence of *HFE* variants varies considerably across geographic regions and ethnic backgrounds. While p.Cys282Tyr is common in Northern Europe, it is rare in Mediterranean, Middle Eastern, and Asian populations. Conversely, p.His63Asp shows a broader distribution and is frequently detected in heterozygous form. These population-specific differences have important implications for genetic screening strategies and clinical interpretation of *HFE* test results.^4^^,^^5^

Data regarding *HFE* variant frequencies in the Turkish population remain limited and are often derived from small cohorts.^6^ Given Türkiye’s unique geographic position and genetic diversity, large-scale population data are essential. Recent global initiatives, such as the Genome Aggregation Database (gnomAD), have further elucidated these disparities, revealing that while the p.Cys282Tyr variant remains highly localized to Northern Europe, other variants like p.His63Asp exhibit a much more ubiquitous global footprint, particularly across Mediterranean and Middle Eastern corridors.^3^^,^^4^^,^^7^ The present study aims to characterize the distribution of *HFE* gene variants in a large Turkish cohort, focusing on genotype and allele frequencies.

## Material and Methods

### Study Population

This retrospective study included 643 consecutive patients who underwent *HFE* gene sequencing at the Medical Genetics Laboratory of Ankara Etlik City Hospital between 2022 and 2025. Patients were referred for genetic testing due to suspected iron metabolism disorders or as part of differential diagnostic evaluation. In general, patients are referred to testing based on laboratory findings such as elevated transferrin saturation (TS), increased serum ferritin concentration, and higher hemoglobin, mean corpuscular hemoglobin, and mean corpuscular volume. However, in some cases, investigations are initiated because of clinical manifestations, including weakness and chronic fatigue; abdominal pain and weight loss; arthropathy (particularly involving the metacarpophalangeal joints, hips, and knees); diabetes; hepatomegaly; cardiomyopathy and arrhythmias; and a progressive increase in skin pigmentation. This study was approved by the Local Ethical Committee of Etlik City Hospital in Ankara/Türkiye (AEŞH-BADEK1-2025-691).

### Genetic Analysis

Genomic DNA was extracted from peripheral blood samples using standard procedures. Sequencing of the *HFE* gene coding regions and exon–intron boundaries was performed using a validated sequencing method (Seqline *HFE *Gene Sequencing Kit, EYSMedikal-Ankara, Türkiye) 3500xl DNA sequencer (Applied Biosystems, Life Technologies Corporation, California, USA). Detected variants were annotated according to Human Genome Variation Society nomenclature. The evaluation of variations adhered to the recommendations outlined in the 2015 American College of Medical Genetics Standards and Guidelines and published literature.^8^

### Statistical Analysis

Descriptive statistical analyses were performed. Genotype and allele frequencies were calculated and expressed as absolute numbers and percentages. The analysis was restricted to genetic frequency data; biochemical and clinical parameters were not included. To estimate cumulative variant prevalence, genome data were derived from 3 primary sources: Turkish Genome Project (TGP) (https://tgd.tuseb.gov.tr/), Turkish Variome (TV) data on the Franklin Genoox server, and the gnomAD.

## Results

### Demographic Characteristics

The study cohort consisted of 643 individuals (190 female, 453 male) with available age data. The mean age of the participants was 46.8 ± 16.9 years (standard deviation), with a median age of 49 years. The age range extended from 6 months to 86 years.

The distribution of ages demonstrated a broad representation across pediatric, adult, and elderly age groups, with the majority of individuals clustered in the middle-aged adult range. This wide age distribution supports the representativeness of the cohort for population-based *HFE* variant frequency analysis.

Age distribution analysis was performed for individuals carrying at least 1 disease-associated *HFE* variant in the heterozygous or compound heterozygous state. This subgroup consisted of 219 patients. The mean age of variant carriers was 46.1 years, with ages ranging from 6 months to 80 years. The age distribution demonstrated a wide span across pediatric and adult age groups, with the majority of individuals clustered in the adult population.

### Genotype Distribution

A total of 643 individuals who underwent *HFE* gene sequencing were included in the analysis. Of these, 424 patients (65.9%) showed a wild-type genotype, with no pathogenic or likely pathogenic variants of unknown significance detected.

The most frequent variant identified in the cohort was c.187C>G (p.His63Asp). Heterozygous H63D was observed in 166 patients (25.8%), while homozygous H63D was detected in 32 patients (4.9%). Overall, H63D was present in 199 individuals (30.9%) either in heterozygous, homozygous, or compound heterozygous states.

The c.845G>A (p.Cys282Tyr, C282Y) variant was considerably less frequent. Heterozygous p.Cys282Tyr was identified in 7 patients (1.08%), and homozygous p.Cys282Tyr in 4 patients (0.6%). In addition, 1 patient (0.1%) carried the p.Cys282Tyr/H63D compound heterozygous genotype.

Rare *HFE* variants were detected in a small number of individuals. These included compound heterozygous genotypes involving H63D and other variants: c.211C>T (p.Arg71*), c.340+1G>A, and c.208C>A (p.Pro70Thr), each identified in 1 patient (0.1%). Additionally, 1 patient carried homozygous H63D in combination with heterozygous p.Arg224Trp. The distribution of *HFE* genotypes among patients is presented in Table 1.

### Allele Frequencies

Allele frequency analysis was performed based on a total of 1286 alleles, including wild-type alleles. The most prevalent variant allele was c.187C>G (p.His63Asp, H63D), with an allele count of 236, corresponding to an allele frequency of 18.3%. This frequency was higher than those reported in the 1000 Genomes Project (TGP; 9.3%) and Türkiye Variome (TV; 12.2%), and was comparable to the frequency reported in gnomAD (10.82%).

The c.845G>A (p.Cys282Tyr, C282Y) variant showed a markedly lower frequency in the cohort, with an allele count of 16 and an allele frequency of 1.2%. This frequency was higher than those reported in TGP (0.4%) and TV (0.32%), but substantially lower than the gnomAD reference frequency (3.38%).

Rare *HFE* variants were detected at very low frequencies. The c.670C>T (p.Arg224Trp) variant was identified with an allele count of 2 (0.15%), a frequency comparable to TGP (0.3%) and higher than that reported in gnomAD (0.014%). Single alleles of c.211C>T (p.Arg71*), c.340+1G>A, c.208C>A (p.Pro70Thr), and c.499C>T (p.Leu167=) were each observed once.

Overall, the allele frequency analysis demonstrated a predominance of the H63D variant, while C282Y and S65C variants were relatively uncommon, and non-classical *HFE* variants occurred at very low frequencies in this cohort. The distribution of *HFE* variant alleles identified in the cohort is summarized in Table 2.

## Discussion

In this large single-center retrospective cohort, a comprehensive overview of *HFE* genotype and allele frequencies are provided, highlighting a population-specific distribution pattern that differs substantially from that observed in European populations. The most striking finding of the study is the relative scarcity of the p.Cys282Tyr homozygosity, accompanied by the predominance of the p.His63Asp both at the genotype and allele levels. These results reinforce the notion that the genetic architecture of HH is strongly population dependent.

When compared with available Turkish and regional datasets, the findings are largely consistent with previously reported patterns. Studies conducted in Turkish cohorts and neighboring populations have consistently demonstrated a low prevalence of p.Cys282Tyr and a relatively higher frequency of p.His63Asp.^9^^,^^10^ These observations are further supported by regional genomic datasets, including the TV and gnomAD Middle Eastern subsets, which similarly indicate that H63D is the predominant *HFE* variant in these populations, whereas C282Y remains uncommon. Together, these data reinforce the validity of the findings and highlight the stability of this distribution pattern across different Turkish cohorts.

The p.His63Asp variant constituted the majority of detected *HFE* alterations in this cohort, with an allele frequency of 18.3% and presence in approximately one-third of individuals when heterozygous, homozygous, and compound heterozygous states were considered. These findings support the notion that H63D is a more common polymorphic variant in populations of the Eastern Mediterranean and Middle Eastern regions, rather than a mutation confined to Northern European ancestry groups.^6^ The clinical significance of the *HFE* c.187C>G (p.His63Asp, H63D) variant remains a subject of ongoing debate. While H63D is widely distributed across different populations, its penetrance is substantially lower than that of the p.Cys282Tyr (C282Y) variant, and isolated heterozygosity for H63D is generally not sufficient to cause classical HH. However, homozygosity for H63D has been associated with mild to moderate iron overload in a subset of individuals, particularly in the presence of additional genetic or environmental modifiers. Importantly, compound heterozygosity for H63D and C282Y has been shown to increase the risk of iron accumulation compared with either variant alone, although clinical expression remains variable.^11^ These observations suggest that H63D functions primarily as a disease modifier rather than a fully penetrant pathogenic variant.

In contrast, the C282Y variant recognized as the principal pathogenic mutation underlying classical HH in Northern Europeans was rare in this cohort. The p.Cys282Tyr variant is known to have originated in Northern Europe and shows a clear north-to-south frequency gradient across the continent. High carrier rates and homozygosity frequencies have consistently been reported in populations of Celtic or Northern European ancestry, whereas the variant becomes increasingly rare toward Southern Europe, the Middle East, and Asia.^4^ Homozygosity for C282Y was observed in less than 1% of individuals, and compound heterozygosity with H63D was detected in only a single case. The observed allele frequency of C282Y (1.2%) was markedly lower than that reported in gnomAD and is consistent with previous reports indicating that this variant is uncommon in Mediterranean, Middle Eastern, and Asian populations.^12^

Although this observation has been suggested in previous smaller studies, the findings support it in a larger clinical cohort.^6^^,^^10^ In a previous study conducted among blood donors, the C282Y mutation was not detected in any of the 286 alleles examined, including 172 alleles from 86 blood donors with TS ≥45% and 114 alleles from 57 healthy controls with TS <45%. In contrast, the H63D variant was relatively common, with heterozygosity observed in 17.1% of individuals and homozygosity in 7.7%, yielding an overall H63D mutation frequency of 24.8%.^13^ As previously discussed, the controversial clinical impact of the H63D variant raises the consideration that non-*HFE* genetic causes should be taken into account in Turkish patients with clinical hemochromatosis.

Rare and non-classical *HFE* variants, including p.Arg224Trp, p.Arg71*, splice-site variant c.340+1G>A, and p.Pro70Thr, were detected only sporadically and exclusively in heterozygous or compound heterozygous states. Their extremely low frequencies are in line with existing population databases and suggest that these variants contribute minimally to the overall genetic burden of *HFE*-related iron metabolism disorders at the population level. Nevertheless, their identification underscores the value of full-gene sequencing approaches over targeted variant testing, particularly in genetically diverse populations.

From a broader population genetics perspective, the Turkish population occupies a unique position at the crossroads of Europe, the Middle East, and Central Asia. Historical and genetic evidence indicates that modern Anatolian populations are shaped by complex admixture events, including indigenous Anatolian groups, Middle Eastern populations, and Turkic migrations from Central Asia beginning in the early medieval period. In this context, comparison with Central and East Asian populations is particularly informative. Studies from East Asia and Central Asia consistently report near absence of p.Cys282Tyr and low overall prevalence of *HFE*-related pathogenic genotypes, supporting the idea that p.Cys282Tyr is largely confined to populations of European origin.^14^

The predominance of p.His63Asp heterozygosity observed in the cohort aligns with reports from Asian populations, where p.His63Asp is present at low-to-moderate frequencies but rarely associated with clinically overt iron overload. This observation further supports the hypothesis that p.His63Asp alone has limited pathogenic potential and may act primarily as a modifier rather than a primary disease-causing variant. In populations such as Türkiye, where p.Cys282Tyr is rare, the clinical significance of isolated p.His63Asp detection should therefore be interpreted with caution, particularly in the absence of biochemical evidence of iron overload. The predominance of the H63D variant in populations where C282Y is rare may have important clinical implications. In such settings, detection of H63D, particularly in the heterozygous state, should not be overinterpreted as indicative of clinically significant HH in the absence of supportive biochemical findings. Instead, H63D should be considered a low-penetrance variant or a potential modifier that may contribute to iron overload only in combination with other genetic or environmental factors. This has direct relevance for clinical practice, as reliance on genetic findings alone may lead to overdiagnosis or unnecessary follow-up investigations. Therefore, integration of genetic data with biochemical markers and clinical context remains essential for accurate diagnosis and management.

The findings have important implications for genetic screening strategies. Applying screening algorithms and interpretative frameworks developed for Northern European populations to Turkish or Middle Eastern populations may lead to overdiagnosis or unnecessary follow-up in individuals carrying low-penetrance variants. Population-specific reference data, such as those provided in this study, are essential for accurate risk assessment and appropriate genetic counseling.

Clinically manifest HH is well known to exhibit a marked male predominance, which has been consistently reported across population-based and referral cohorts. This sex difference is largely attributed to physiological iron loss in females through menstruation and pregnancy, leading to delayed iron accumulation and later clinical presentation compared with males.^1^ In line with this established pattern, the cohort also demonstrated a clear male predominance among individuals undergoing *HFE* genetic testing. Although the present study was not designed to assess sex-specific penetrance or clinical severity, the observed distribution is consistent with previous reports indicating that males are more frequently referred for evaluation of iron overload and are more likely to reach diagnostic thresholds at earlier ages.

In patients carrying heterozygous variants (predominantly H63D), in those harboring combinations of 2 low-penetrance variants, or in individuals in whom no pathogenic *HFE* variant is detected, further evaluation of large deletions or duplications in the *HFE*, as well as analysis of other genes associated with HH, may contribute to elucidating the causative factors of the disease. In addition, consideration of environmental and acquired factors may provide complementary insights into disease expression. Such approaches could facilitate the generation of more comprehensive data relevant to HH in the Turkish population and enrich the existing literature.

### Limitations

In this study, the *HFE* gene was not evaluated for potential copy number alterations using gold-standard methods. Therefore, the contribution of large deletions or duplications to this disease group may be clarified by future investigations. Furthermore, as a retrospective analysis conducted at a single tertiary center, the cohort may reflect a degree of referral bias and may not fully represent the general Turkish population. The analysis focused exclusively on genetic frequency data; the absence of concurrent biochemical parameters (such as serum ferritin or TS) and clinical phenotypes prevents the assessment of genotype-phenotype correlations or the clinical penetrance of the detected variants. The regional or nationwide distribution of the reported variants in Türkiye is not yet well defined. Because genome databases representing the Turkish population included fewer than 10 000 alleles at the time of this study, reliable estimation of carrier frequencies for these variants may be improved in future additions. Establishing a large-scale, nationally representative genomic database would therefore be a valuable resource for the field. Future studies integrating genetic and phenotypic data are needed to better understand genotype–phenotype correlations in the Turkish population.

In conclusion, the *HFE* genetic profile of the Turkish population reflects a pattern intermediate between European and Middle Eastern-Asian populations.^3^ The low frequency of p.Cys282Tyr and the predominance of p.His63Asp heterozygosity underscore the importance of considering historical population movements and genetic background when interpreting *HFE* test results. These findings emphasize the need for population-specific guidelines in the genetic evaluation of HH.

## Figures and Tables

**Table 1.** Distribution of *HFE* Genotypes Among 643 Patients

***HFE* Genotype**	**Number of Patients (n)**	**Percentage (%)**
Wild-type (no variant detected)	424	65.9
c.187C>G p.His63Asp (heterozygous)	166	25.8
c.187C>G p.His63Asp (homozygous)	32	4.9
c.845G>A p.Cys282Tyr (heterozygous)	7	1.08
c.845G>A p.Cys282Tyr (homozygous)	4	0.6
c.845G>A p.Cys282Tyr / c.187C>G p.His63Asp (compound heterozygous)	1	0.1
c.187C>G p.His63Asp (homozygous)/ c.670C>T p.Arg224Trp (heterozygous)	1	0.1
c.187C>G p.His63Asp/ c.211C>T p.Arg71* (compound heterozygous)	1	0.1
c.187C>G p.His63Asp/ c.340+1G>A (compound heterozygous)	1	0.1
c.187C>G p.His63Asp/ c.208C>A p.Pro70Thr (compound heterozygous)	1	0.1
c.499C>T p.Leu167= (heterozygous)	1	0.1
c.670C>T p.Arg224Trp (heterozygous)	1	0.1
c.193A>T p.Ser65Cys (heterozygous)	1	0.1
Total	643	100

**Table 2.** Allele Frequencies of the *HFE* Variants Identified in the Cohort. Total Alleles; 1286 (Including Wild Types)

**Variant**	**Allele Count**	**Allele Frequency **	**TGP**	**TV**	**gnomAD**
c.187C>G p.His63Asp	236	0.183	0.093	0.122	0.1082
c.845G>A p.Cys282Tyr	16	0.012	0.004	0.0032	0.03377
c.670C>T p.Arg224Trp	2	0.0015	0.003	0.00059	0.0001449
c.211C>T p.Arg71*	1	0.00077	—	—	0.000009294
c.340+1G>A	1	0.00077	—	0.00058	0.000001239
c.208C>A p.Pro70Thr	1	0.00077	—	—	0.000002478
c.499C>T p.Leu167=	1	0.00077	—	—	0.000003098
c.193A>T p.Ser65Cys	1	0.00077	0.006	0.002	0.01364

gnomAD, Genome Aggregation Database; TGP, Turkish Genome Project; TV, Turkish Variome.

## Data Availability

The data that support the findings of this study are available on request from the corresponding author.
